# Confounders, diagnostic accuracy, and reproducibility in cardiovascular magnetic resonance-feature tracking-derived left atrial strain: A Berlin Research Network for cardiovascular magnetic resonance multi-software, multi-site comparison

**DOI:** 10.1016/j.jocmr.2026.102692

**Published:** 2026-01-15

**Authors:** Leo Dyke Krüger, Leonhard Grassow, Jan Gröschel, Johanna Kuhnt, Edyta Blaszczyk, Maximilian Müller, Ralf Felix Trauzeddel, Teodora Chitiboi, Jeanette Schulz-Menger, Maximilian Fenski

**Affiliations:** a Charité – Universitätsmedizin Berlin, corporate member of Freie Universität Berlin and Humboldt-Universität zu Berlin, ECRC Experimental and Clinical Research Center, Berlin, Germany; b Working Group on Cardiovascular Magnetic Resonance, Experimental and Clinical Research Center, a joint cooperation between Charité Medical Faculty and the Max-Delbrück Center for Molecular Medicine, Berlin, Germany; c DZHK (German Centre for Cardiovascular Research), partner site Berlin, Berlin, Germany; d HELIOS Clinics Berlin Buch, Department for Cardiology and Nephrology, Berlin, Germany; e Deutsches Herzzentrum der Charité - Department of Cardiology, Angiology and Intensive Care Medicine, Berlin, Germany; f Department of Anesthesiology and Intensive Care Medicine, Charité Campus Benjamin Franklin, Berlin, Germany; g Siemens Healthineers AG, Hamburg, Germany; h Radcliffe Department of Medicine, Division of Cardiovascular Medicine, University of Oxford, Oxford, UK

**Keywords:** Normal ranges, Software, Influencing factors, Deformation

## Abstract

**Background:**

Cardiovascular magnetic resonance-feature tracking (CMR-FT) derived left atrial global longitudinal strain (LA-GLS) has prognostic relevance, even in the early stages of cardiovascular diseases. Identifying technical and subject-related confounders is essential for ensuring comparability across sites and for reliably distinguishing healthy from pathological conditions. This study aimed to evaluate the influence of post-processing software and subject-related factors on CMR-FT-derived LA-GLS, diagnostic accuracy, and to evaluate inter-site reproducibility.

**Methods:**

This study included 149 healthy individuals and 40 patients with atrial fibrillation (AF; 19 persistent, 21 paroxysmal) from a single site. A subgroup of 18 traveling volunteers underwent CMR at four different sites. All participants underwent CMR in sinus rhythm. LA-GLS was assessed using three post-processing software packages (CVI42, TrufiStrain Research Prototype, Medis). Mixed models with repeated measures were applied to evaluate the effect of software, site, and subject-related factors on LA-GLS components. ROC curve analysis was used to assess diagnostic accuracy across software in distinguishing healthy controls from AF patients.

**Results:**

All GLS components differed across post-processing software (p<.001). Reservoir and contractile GLS were lowest in CVI42 (23.9% ± 3.3%, 9.9% ± 2.2%), followed by TrufiStrain (27.4% ± 6.3%, 15.0% ± 4.8%) and Medis (45.4% ± 9.7%, 20.3% ± 5.7%). Conduit GLS was lowest in TrufiStrain (12.4% ± 4.8%), followed by CVI42 (16.3% ± 4.5%) and Medis (25.1% ± 8.2%). Among traveling volunteers, LA-GLS values were consistent across sites when the same software was used. Across all software, reservoir GLS negatively correlated with age. Diagnostic accuracy was comparable across software packages (AUC for reservoir strain: CVI: 0.81 [0.69–0.90], TrufiStrain 0.76 [0.64–0.88], Medis: 0.84 [0.72–0.94]).

**Conclusion:**

Post-processing software is a significant confounder in CMR-FT-based LA-GLS analysis and age substantially influences LA-GLS. LA-GLS demonstrates excellent inter-site reproducibility when analyzed with the same software and offers comparable diagnostic accuracy across platforms.

## Graphical abstract

**Figure:**
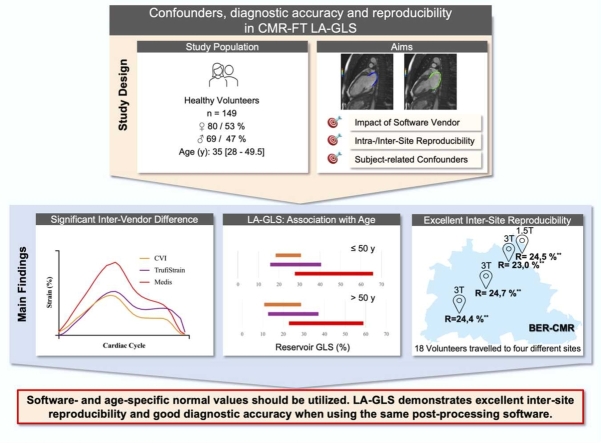
Confounders and reproducibility in CMR-feature tracking-derived LA-GLS. Investigation of normal value ranges using three different types of post-processing software. Significant differences were evident between software vendors. Analysis showed excellent inter-site reproducibility. The association for age is shown for reservoir GLS. Age-group-specific normal value ranges for each GLS component are detailed in the paper. * Research Prototype; ** Reservoir GLS; *BER-CMR* Berlin Research Network for Cardiovascular Magnetic Resonance, *CMR-FT* cardiovascular magnetic resonance-feature tracking, *GLS* Global longitudinal strain, *LA* left atrium, *y* Years

## 1 Introduction

Cardiovascular magnetic resonance-feature tracking (CMR-FT) applied to the left atrium (LA) provides a quantitative analysis of LA global longitudinal strain (LA-GLS), a parameter increasingly recognized for its prognostic significance in cardiovascular diseases and response to therapies [1], [2], [3], [4], [5], [6], [7], [8], [9]. Unlike global measures such as ejection fraction, which may not fully reflect the complexity of cardiac function, GLS analysis offers a more comprehensive understanding of regional myocardial function and deformation [10], [11]. However, research into LA-GLS is still developing, with gaps in standardization and reproducibility across sites. Variables such as age, sex, and differences in post-processing protocols may induce variations in CMR-FT-derived LA-GLS measurements [12], [13], [14], [15], [16]. Despite the growing interest in LA-GLS, particularly as a prognostic marker, the lack of inter-software comparability and consensus on standard post-processing protocols hinder broader clinical adoption. In addition, a recent meta-analysis indicated regional differences in LA-GLS components among Asian, North American, and European studies [12]. However, these findings were limited as only a single European study was available for inclusion. Furthermore, although previous studies have demonstrated good inter- and intra-reader agreement [12], [13], [15], the inter- and intra-site reproducibility of CMR-FT-derived LA-GLS remains unestablished. This necessitates comprehensive evaluation to ensure diagnostic accuracy and reliability in multi-center studies and longitudinal investigations. In the past, efforts were made to establish normal values in atrial and ventricular size and function measurements in multi-center studies [17]. However, LA-GLS was not included in this prior work.

This study aimed to fill these gaps by assessing the impact of post-processing software and subject-specific confounders on LA-GLS in healthy individuals of European ancestry. It also presents age and software-specific normal values and assesses the diagnostic performance and inter-/intra-site reproducibility of LA-GLS across software to support its broader application.

## 2 Methods

### 2.1 Study design

This study retrospectively analyzed CMR images from a cohort of prospectively enrolled healthy individuals aged ≥18 years. All CMR data acquired as part of prospective studies at our institution between June, 2011 and May, 2023 were screened for examinations of healthy volunteers. To evaluate the diagnostic accuracy of LA-GLS across software platforms in distinguishing healthy individuals from patients with confirmed atrial disease, we also included 40 patients with atrial fibrillation (AF; n = 21 paroxysmal, n = 19 persistent) who underwent CMR in sinus rhythm prior to their first pulmonary vein ablation. All included studies had prior approval from the ethics committee of Charité University Medicine Berlin and complied with institutional standards and the Declaration of Helsinki. Retrospective data analysis was also approved by the same ethics committee.

### 2.2 Study population

Participants were considered healthy if they met the following criteria: 1. No history of cardiovascular disease, chronic inflammatory skin or autoimmune disorders, pulmonary-, neurological-, or gastrointestinal conditions, diabetes, malignancy, or use of systemic medication. 2. No electrocardiographic (ECG) abnormalities; and 3. No pathological findings on CMR. Self-reported questionnaires were used to assess smoking status, medical history, and medication use. A 12-channel ECG was performed prior to scanning to exclude conduction abnormalities. Height, weight, blood pressure, and heart rate were documented. The diagnosis of AF was established based on 12-lead ECG or Holter-monitoring, in accordance with European Society of Cardiology guidelines [18]. All AF patients were referred for pulmonary vein isolation as treatment for symptomatic AF. To account for the association between LA-GLS and age, only the 20 oldest healthy individuals with acceptable tracking quality across all software were included for assessment of diagnostic accuracy. Participants with CMR contraindications were excluded. The cohort was stratified by biological sex (female/male).

### 2.3 Image acquisition

All scans were conducted at either 1.5 Tesla (T) (AvantoFit, Siemens Healthineers, Forchheim, Germany) or 3T (SkyraFit, Verio and PrismaFit, Siemens Healthineers, Forchheim, Germany) in sinus rhythm. Patients with AF and arrhythmias during image acquisition were excluded from the analysis. A subgroup of 18 traveling healthy participants underwent CMR at four sites within the Berlin Research Network for CMR (BER-CMR [19]): One 1.5T scan at site 1, 3T scans at sites 2–4, and a repeat scan at site 4. The CMR protocol included anatomical localizers and balanced steady-state free precession (bSSFP) cine imaging in standardized long axis (LAx) views. Cine images with 30 phases were acquired in two-, three, four-chamber, and right ventricular views (2CV, 3CV, 4CV, RVV), in line with Schmidt-Rimpler et al. for reliable strain analysis [14]. ECG triggering with retrospective gating was used. Sequence parameters are detailed in the Supplemental Methods 1.

### 2.4 Image analysis

All CMR images were analyzed for cardiac function and LA-GLS. Cardiac size and function was assessed using CVI42 software (Circle Cardiovascular Imaging, version 5.13.7, Calgary, Canada), in accordance with current standards [20]. Volumetric quantification was performed using the biplane Simpson method [21].

### 2.5 CMR-FT LA-GLS analysis

**Fig. 1:**
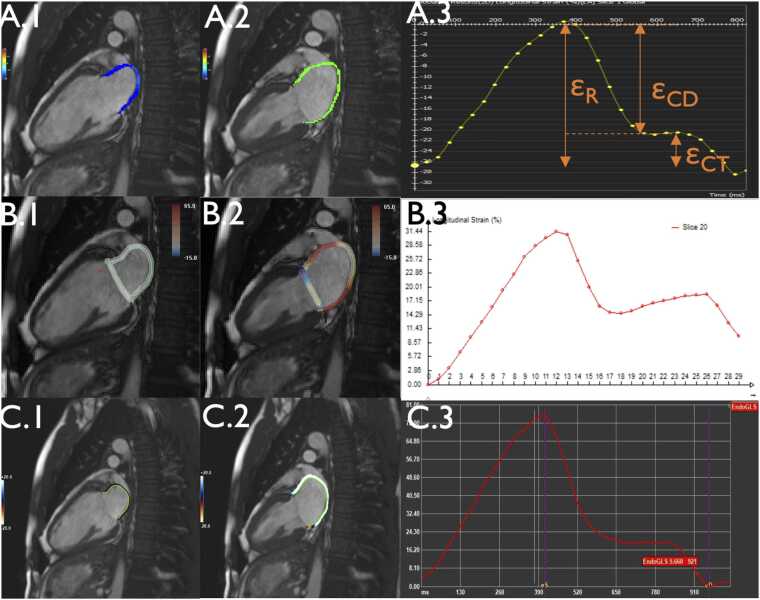
Left atrial contours and GLS curves, (A) Endocardial contours for 2-chamber view atrial end-systole (A.1) and atrial end-diastole (A.2) plus GLS curve (A.3) in CVI42. (B) Endocardial contours 2-chamber views atrial end-systole (B.1) and atrial end-diastole (B.2) plus GLS curve (B.3) in TrufiStrain Research Prototype. (C) Endocardial contours 2-chamber views atrial end-systole (C.1) and atrial end-diastole (C.2) plus GLS curve (C.3) in Medis. *ε_R_* Reservoir GLS, *ε_CD_* Conduit GLS, *ε_CT_* Contractile GLS, *GLS* Global longitudinal strain

LA-GLS was assessed using three post-processing software tools employing CMR-FT based on optical flow methods [9], [10]: Software 1) CVI42 (Circle Cardiovascular Imaging, version 5.13.7, Calgary, Canada); Software 2) TrufiStrain 3.0 Research prototype (Siemens Healthineers, Forchheim, Germany)^1^; Software 3) Medis (Medical Imaging Systems, version 4.0.62.4, Leiden, Netherlands). Images were analyzed by a single experienced CMR reader (L.D.K.), with regular supervisions held with a second experienced CMR reader (M.F.). GLS analysis was performed manually in a standardized procedure: Contours were drawn along the subendocardial boarder of the LA in 2CV and 4CV, with pulmonary veins and the LA appendage excluded. The same views were used across software. In CVI42 and TrufiStrain, contours were propagated from the first cardiac phase; in Medis propagation was based on end-systolic and end-diastolic images. Fig. 1 provides examples of contours and resulting LA-GLS curves. View Supplemental Methods 2 for further details. All cases were individually inspected for tracking quality and contours were adjusted if necessary. Cases with poor tracking—such as failure to follow the mitral annulus—were excluded (Examples in Fig. S1). Only complete measurement pairs, including 2CV and 4CV with satisfactory image and tracking quality, were included in the final analysis. The resulting LA-GLS curve typically exhibits two peaks as follows: Reservoir GLS (peak during ventricular systole), and contractile GLS (peak value during the subsequent plateau phase). The difference between these two peaks defines the conduit GLS [12], [14]. Final strain values were calculated as the average of 2CV and 4CV. Individual LA-GLS curves were exported as comma-separated values (CSV) files. Reservoir, conduit, and contractile components were extracted using an in-house MATLAB script (version R2023b Update 3, MathWorks, Natick, Massachusetts). For CVI42 and Medis, strain curves were referenced to the point of LA end systole. TrufiStrain, by default, uses the first cine image as its reference.

### 2.6 Inter- and intra-site variability

Variability was assessed across different sites and between two repeated scans at the same site. Of all included healthy subjects (n = 149), a subgroup of 18 traveling volunteers underwent CMR at four different sites within the BER-CMR research network, with a median interval of 8 days, to evaluate inter-site reproducibility. To assess combined intra-site and intra-reader variability, two consecutive scans were performed at site 4, separated by a 15-minute interval, including subject repositioning and reacquisition of cine data.

### 2.7 Statistical analysis

Analyses were conducted using SAS (SAS Institute Inc., version 9.4, Cary, North Carolina), SPSS (IBM, version 29.0.1.1, Armonk, New York) and GraphPad Prism 10 (GraphPad Software, version 10.1.1, San Diego, California). Continuous parameters were expressed as mean ± standard deviation (SD) if normally distributed, or as median with interquartile range otherwise. Categorical variables were presented as counts and percentages. Normality was assessed using Shapiro-Wilk test and QQPlots. Sex-based comparisons of baseline characteristics were performed using the Student’s t-test or Mann–Whitney-U test, as appropriate. Associations between potential confounders and GLS components were examined using a mixed model with repeated measures and post hoc intergroup comparisons. Fixed factors in the mixed model included software, field strength, scanner type, BMI, sex, age, heart rate (HR), systolic- and diastolic blood pressure. Repeated measures per subject were accounted for by including subject as a repeated factor with compound symmetry as covariance structure. Backward selection was used to identify relevant factors in the model, removing the factor with the weakest effect until all remaining factors in the model had a p-value of <0.05 in the type III test for fixed effects.

The same model with site added as a fixed factor was used to assess inter-site reproducibility. Only data sets with acceptable tracking quality across all three software platforms were included in post hoc comparisons. Fisher’s exact test was used to assess whether exclusion rate differed between 1.5T and 3T scans. Intra-site and intra-reader agreement was evaluated using Bland-Altman analysis. Associations between continuous variables and GLS components were further analyzed using Pearson or Spearman correlation, as appropriate. ROC curve analyses were applied to assess diagnostic accuracy. Significance levels were set at p =.05. Due to the exploratory nature of the study, no adjustment for multiple testing was applied. Normal value ranges were calculated as mean ± 2 SD.

## 3 Results

### 3.1 Baseline characteristics

Of 162 volunteers meeting the inclusion criteria, 13 were excluded due to poor image quality. The final cohort comprised 149 healthy participants (53.7% (80/149) female) and 40 patients with AF (21 paroxysmal, 19 persistent). Baseline characteristics for healthy individuals are summarized in Table 1. Males showed higher left ventricular (LV) end-diastolic volume, stroke volume, LV mass indexed to BSA, and LA max. area (Table 2). No significant sex differences were observed for LA function. Satisfactory tracking quality in both 2CV and 4CV was achieved in n = 147 for CVI42, n = 132 for TrufiStrain and n = 133 for Medis. Reasons for exclusion specific to each software are detailed in Fig. S1.

**Table 1:** Baseline characteristics of healthy individuals

	Overall (n = 149)	Female (n = 80)	Male (n = 69)	p-value*
Age (y)	35 [28–49.5]	38 [30–51.8]	33 [28–49.5]	.026
Height (cm)	174±9	168±6	181±7	<.001
Weight (cm)	72±13	66±9	79±13	<.001
BMI (kg/m^2^)	23.4 [21.5–25.6]	23.1 [21.2–25.5]	24.0 [21.9–25.6]	.251
BSA (m^2^)	1.86±0.19	1.76±0.13	1.99±0.17	<.001
HR (beats/min)	67 [63–75]	69 [64–77]	68 [61–75]	.219
RR_sys_ (mmHg)	123±13	122±15	124±10	.401
RR_dia_ (mmHg)	74±9.3	74±10	74±8	.634
MRI scanner (1.5 T/3 T)	123/26	71/9	52/17	.031

Values are mean ± standard deviation, median [Inter-Quartile-Range] or absolute (n).

*BMI* body mass index; *BSA* body surface area; *HR* heart rate, *MRI* magnetic resonance imaging, *RR_sys_* systolic blood pressure, *RR_dia_* diastolic blood pressure, *for comparison between female and male participants

**Table 2:** Baseline cardiac function of healthy individuals

	Overall (n = 149)	Female (n = 80)	Male (n = 69)	p-value
LV EF (%)	62.7±4.8	63.4±4.9	61.8±4.6	.042
LV EDV-I (mL/m^2^)	85.9±15.2	79.7±12.9	93.2±14.4	<.001
LV SV-I (mL/m^2^)	53.7±9.6	50.5±8.5	57.5±9.4	<.001
LV M-I (g/m^2^)	50.5 [44.8–58.4]	46.2 [39.8–52.5]	56.7 [49.2–66.9]	<.001
LA A_max_ (cm^2^)	21.1 [18.7–23.9]	20.1 [18.4–23.3]	22.3 [19.2–25.3]	.017
LA EF (%)	64.8 [60–69.8]	66.3 [59.3–71.1]	64.5 [60.1–69.0]	.213
LA V_max_ -I (mL/m^2^)	33 [28.1–39.9]	32 [28.4–38.6]	33.6 [27.7–40.4]	.304
LA SV-I (mL/m^2^)	22.4±5.6	22.0±4.8	22.8±6.3	.391

Values are mean ± SD or median [interquartile range].

*LA A_max_* left atrial max. area, *LA EF* left atrial ejection fraction, *LA SV-I* left atrial stroke volume indexed to BSA, *LV EF* Left ventricular ejection fraction, *LA V_max_ –I* left atrial max. volume indexed to BSA, *LV EDV-I* left ventricular end-diastolic volume indexed to BSA, *LV M-I* left ventricular mass indexed to BSA, *LV SV-I* Left ventricular stroke volume indexed to BSA

### 3.2 Post-processing software as a confounder

**Fig. 2:**
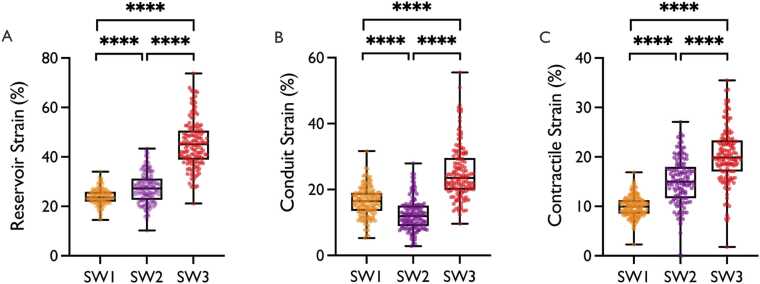
CMR-FT Software Comparison. (A) Left atrial reservoir GLS (B) Left atrial conduit GLS (C) Left atrial contractile GLS. Data for CVI42 is shown in orange, TrufiStrain Research Prototype in purple and Medis in red. Box and Whisker Plots with boxes showing mean and interquartile range, and whisker presenting minimum and maximum values. ****p <.001; *GLS* Global longitudinal strain, *SW 1* CVI42, *SW2* TrufiStrain Research Prototype, *SW 3* Medis, *CMR-FT* Cardiovascular magnetic resonance-feature tracking

Significant differences were observed between all software vendors for all LA-GLS components (p <.001, Fig. 2, Table 3). Reservoir and contractile GLS values were lowest in CVI42, followed by TrufiStrain and Medis. Conduit GLS was lowest in TrufiStrain, followed by CVI42 and Medis. Bland-Altman plots revealed proportional bias with larger inter-software differences at higher GLS values (bias [limits of agreement]; CVI vs. TrufiStrain: −3.59 [7.24 to −14.42], CVI vs. Medis: −21.5 [−4.09 to −38.98], TrufiStrain vs. Medis: −17.8 [0.16 to −35.86]) (Fig. S2-S4). CVI42 showed the lowest overall variance (Reservoir: 11.22%, conduit: 23.07%, contractile: 4.67%) followed by TrufiStrain (Reservoir: 39.82%, conduit: 23.33%, contractile: 23.43%) and Medis (Reservoir: 94.48%, conduit: 67.90%, contractile: 32.38%). No significant difference was found between the global GLS value, provided by CVI and the extracted value at end-systole (24.3 ± 3.21 vs. 23.9 ± 3.35, p =.249).

**Table 3:** Global longitudinal strain values for each software

	CVI	TrufiStrain*	Medis	P-value	Effect size	Confidence interval
Reservoir (%)	23.9±3.3	27.4±6.3	45.4±9.7	<.001	30.75	28.15 to 33.36
Conduit (%)	16.3±4.5	12.4±4.8	25.1±8.2	<.001	6.29	1.37 to 11.22
Contractile (%)	9.9±2.2	15.0±4.8	20.3±5.7	<.001	19.61	14.55 to 24.67

Values are mean ± SD ; *Research prototype. Global longitudinal strain values, P-values, effect sizes and confidence intervals for each software. Strain values are given in %. Effect sizes are unitless.

### 3.3 Inter- and intra-site variability

**Fig. 3:**
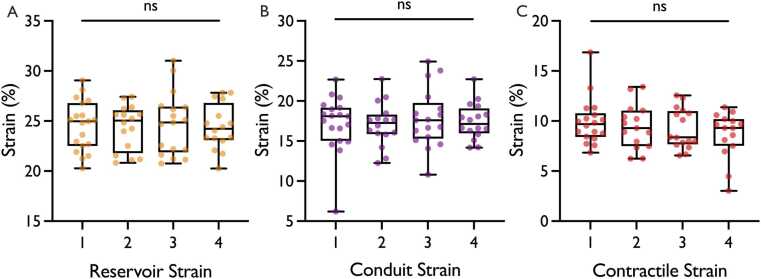
Inter-Site Comparison. (A) Comparison of reservoir GLS (orange) between site 1 to 4 (B) Comparison of conduit GLS (purple) between sites 1 to 4 (C) Comparison of contractile GLS (red) between sites 1 to 4. Box and Whisker Plots with box showing mean and interquartile range, and whisker presenting minimum and maximum values. 1 = site 1 (1.5 T); 2 = site 2 (3 T); 3 = site 3 (3 T); 4 = site 4 (3 T); *GLS* Global longitudinal strain, *ns* not significant

No significant site-related differences were observed in any GLS component when analyzed with CVI42 (reservoir: p =.738; conduit: p =.939; contractile: p=.417; Fig. 3). Bland-Altman plots showed no systematic bias between sites (mean bias = 0.28; maximal limits of agreement [7.52 to −8.09]) when using same software and version (Figs. S5–S7). Intra-site and intra-reader reproducibility was high for repeated scans at the same site using CVI42, with no significant differences and good agreement (p >.389; (bias [limits of agreement]; reservoir: 2.19 [−4.26 to 4.34]; conduit: 2.23 [−4.26 to 4.46]; contractile: 2.08 [3.5 to −4.63], Fig. S8).

### 3.4 Subject-related confounders

#### 3.4.1 Age

**Fig. 4:**
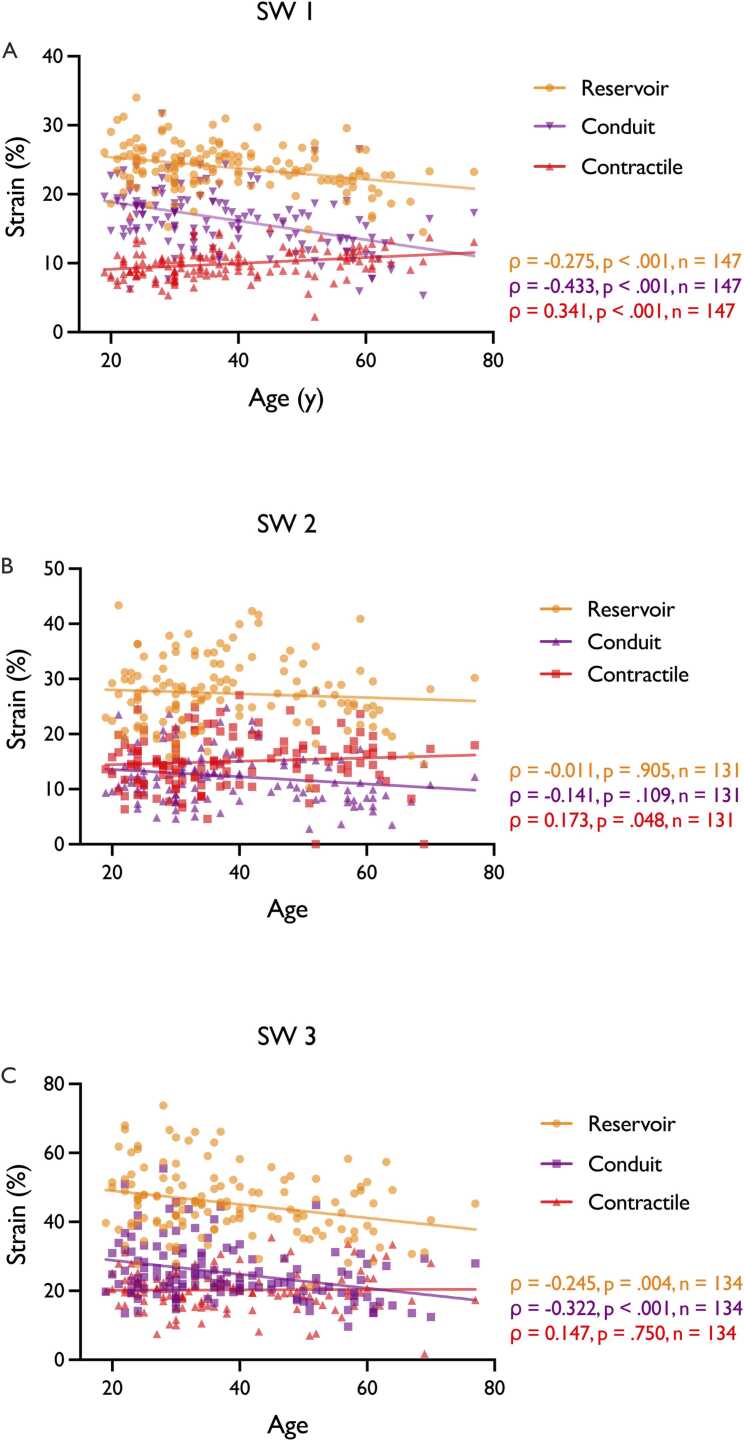
Correlation between LA-GLS and Age. Correlation Analysis. (A) LA-GLS according to age for CVI42 (B) LA-GLS according to age for TrufiStrain Research Prototype (C) LA-GLS according to age for Medis. Reservoir GLS is shown in orange. Conduit GLS is shown in purple. Contractile GLS is shown in red. *GLS* Global longitudinal strain, *ρ* Spearman Correlation Coefficient, *SW 1* CVI42, *SW 2* TrufiStrain Research Prototype, *SW 3* Medis

Age significantly influenced reservoir and conduit GLS (Reservoir: p <.001, effect size −0.11, confidence interval −0.17 to −0.052; Conduit: p <.001, effect size −0.15, confidence interval −0.19 to −0.10), but not contractile GLS (p =.480). On average, reservoir and conduit GLS decreased by 0.11 and 0.15 percentage points per additional year of age, respectively. Negative correlations were found between age and reservoir GLS in CVI42 (ρ = −.275, p <.001) and Medis (ρ = −.205, p =.004) and age and conduit GLS in CVI42 and Medis (ρ = −.433, p <.001 and ρ = −.322, p <.001, respectively, Fig. 4). A positive association between age and LA contractile GLS, was observed in CVI42 and TrufiStrain (ρ =.341, p <.001 and ρ =.173, p =.48, respectively). Age group analysis (≤ 50 vs >50 years) revealed significant differences for reservoir and conduit GLS in all software (p ≤.01). Contractile GLS differed significantly only in CVI42 (p =.01). Age-specific normal values are presented in Table 4. Further analysis is shown in Fig. S9 and Tables S1-S4.

**Table 4:** Normal ranges for LA-GLS according to age for each software

		≤50 years		>50 years	
Software		Mean±SD (CI)	LL – UL^**^	Mean±SD (CI)	LL – UL^**^
CVI	Reservoir (%)	24.5±3.1 (23.9 to 25.1)	18.3–30.7	21.9±3.4 (20.7–23.1)	10.7–28.6
	Conduit (%)	17.3±3.9 (16.5 to 18.0)	9.3–25.2	13.3±4.8 (11.6 to 18.0)	3.7–22.9
	Contractile (%)	9.7±2.1 (9.4 to 10.1)	5.6–13.8	10.5±2.4 (9.6 to 11.3)	5.6–15.3
TrufiStrain*	Reservoir (%)	28.1±6.2 (26.9 to 29.4)	15.7–40.5	24.8±6.1 (22.5 to 27.1)	12.6–37.0
	Conduit (%)	13.0±4.6 (12.1 to 14.0)	3.7–22.3	10.0±4.9 (8.3 to 11.9)	0.3–19.7
	Contractile (%)	15.1±4.7 (14.2 to 16.0)	5.8–24.4	14.7±5.5 (12.7 to 16.8)	3.8–25.5
Medis	Reservoir (%)	46.8±9.6 (44.9 to 48.6)	27.7–65.9	40.8±9.0 (37.3 to 44.2)	22.7–58.8
	Conduit (%)	26.4±8.1 (24.8 - 27.9)	10.2–42.6	20.9±7.5 (18.1 to 23.7)	6–35.8
	Contractile (%)	20.4±5.0 (19.4 to 21.4)	10.3–30.6	19.9±7.7 (17.1 to 22.8)	4.6–35.2

Normal ranges for left atrial-global longitudinal strain (LA-GLS) with the corresponding confidence intervals (CI) as well as lower and upper limits (LL, UL) according to age for each software. Strain values are given in %.

#### 3.4.2 Sex

**Fig. 5:**
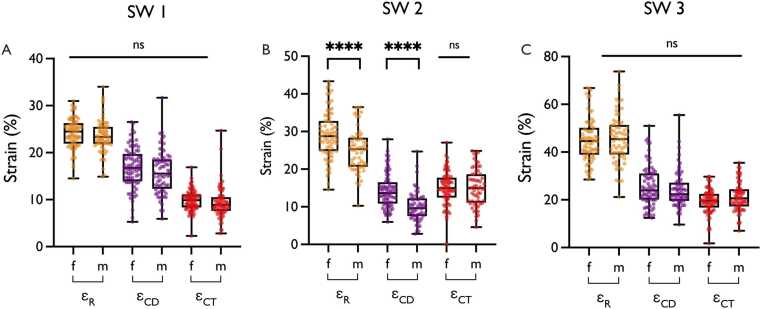
Sex Differences in LA-GLS. (A) Sex comparison for each GLS comparison in CVI42. (B) Sex comparison for each GLS comparison in TrufiStrain Research Prototype. Sex comparison for each GLS comparison in Medis. Box and Whisker Plots with boxes showing mean and interquartile range, and whisker presenting minimum and maximum values. ****p <.001; *ε_R_* Reservoir GSL, *ε_CD_* Conduit GLS, *ε_CT_* Contractile GLS, *f* female, *GLS* Global longitudinal strain, *m* male, *ns* not significant, *SW 1* CVI42, *SW 2* TrufiStrain Research Prototype, *SW 3* Medis, *LA* left atrial

Sex significantly influenced reservoir (p =.024, effect size: 1.9, confidence interval: 0.26 to 3.54) and conduit GLS (p <.001, effect size 2.63, confidence interval: 1.31 to 3.95). On average, females exhibited reservoir and conduit strain values that were 1.9 and 2.63 percentage points higher, respectively. However, post-hoc analysis showed significant differences only in TrufiStrain (p <.001). No sex differences were observed in CVI42 (all p ≥.201) or Medis (all p ≥.123) (Fig. 5).

#### 3.4.3 Heart rate

HR was found to influence conduit GLS (p <.001, effect size 0.15, confidence interval 0.08 to 0.21) with a positive correlation observed between HR and conduit GLS across all software (CVI42: ρ = 0.186; p =.024; TrufiStrain: ρ = 0.222; p =.011; Medis: ρ = 0.378; p <.001) (Fig. S10). On average, conduit GLS increased by 0.15 percentage points per additional beat per minute.

#### 3.4.4 Blood pressure

No significant correlation between blood pressure and LA GLS was found in this study (RR_systolic_ p >.500; RR_diastolic_ p >.149). Tables S1-S3 contain a detailed overview of the results for the respective strain components.

### 3.5 Impact of field-strength

No significant differences in GLS components were observed between 1.5T and 3T scans across all software (all p ≤.590). However, a greater number of 3T cases were excluded due to poor tracking (CVI: 0/123 at 1.5T vs. 2/26 at 3T, p <.001; TrufiStrain: 6/123 at 1.5T vs. 11/26 at 3T, p <.001; Medis: 10/123 at 1.5T vs. 6/26 at 3T, p =.07).

### 3.6 Sensitivity analysis

Sensitivity analysis, limited to participants with adequate tracking quality across all three software vendors (n = 122), confirmed the main findings regarding the influence of software, age, sex, and HR (Figs. S11–S14).

### 3.7 Diagnostic accuracy

**Fig. 6:**
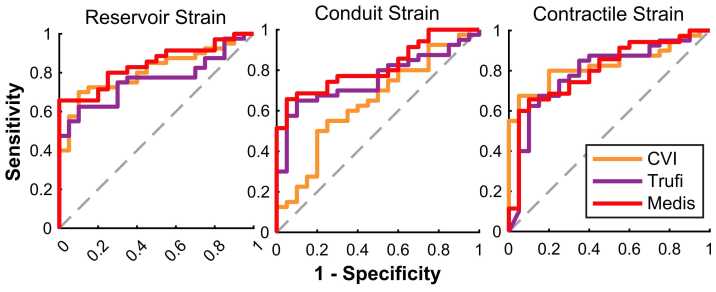
Diagnostic accuracy comparison. ROC curve analysis for calculation of diagnostic accuracy for all strain components

All software demonstrated good diagnostic accuracy in distinguishing healthy individuals from patients with atrial fibrillation when using reservoir strain (Area under the curve (AUC): CVI42 0.81 [0.69–0.90], TrufiStrain 0.76 [0.64–0.88], Medis 0.84 [0.72–0.94]) and contractile strain (AUC: CVI42 0.83 [0.72–0.92], TrufiStrain 0.79 [0.66–0.91], Medis 0.80 [0.67–0.91]). See Fig. 6 and Table S5 for ROC curve analyses. Baseline characteristics of AF patients and detailed strain measurement results are provided in Table S6, S7, and S8.

## 4 Discussion

This study set out to investigate three important aspects of LA-GLS analysis as follows: (1) The impact of post-processing software on strain measurements and diagnostic accuracy, (2) inter- and intra-site reproducibility, and (3) the role of subject-related confounders. In a large cohort of healthy individuals of European ancestry, we demonstrated that both software choice and subject characteristics significantly impact CMR-FT-derived LA-GLS measurements. First, LA-GLS values differed significantly between software vendors. However, all software achieved comparable diagnostic accuracy in differentiating healthy individuals from AF patients. Second, LA-GLS showed excellent reproducibility across different sites and repeated scans when analyzed using the same software. Third, age was significantly associated with LA-GLS, with notable differences between individuals aged ≤ 50 and > 50 years. These findings are discussed in more detail below.

### 4.1 Software as a confounder

In this study, reservoir and contractile GLS were lowest in CVI42, followed by TrufiStrain and Medis. This lack of inter-software comparability has been described previously and remains a challenge [12], [13]. Our finding of proportional bias—where differences between software increase at higher GLS values—mirrors previous observations [13] and may relate to the underlying geometrical models, which may inadequately capture LA motion [13]. Subsequently, the relative error grows with the magnitude of LA-GLS values, which may reflect separate physiological aspects of LA function. As software vendors do not fully disclose their tracking and strain computation methods, the precise reasons remain speculative. However, it seems inter-software variability is mainly caused by proprietary differences rather than differences in contouring [22]. Further efforts towards standardization or transformation between software vendors for LA GLS are therefore urgently needed [23].

In practice, when comparing strain values across platforms, vendor-specific normal ranges should be taken into consideration to identify impaired LA function. However, this only allows identification of worsening or improvement beyond the limits of normal on longitudinal scans. Other approaches, such as z-scoring, may allow for better comparability across software but require further study and validation.

### 4.2 Inter-study variations

The mean reservoir GLS in CVI42 (23.9%) aligns with prior studies, which reported values ranging from 16% to 33% [12], [15], [24], [25]. Other studies reported higher reservoir values (39.1–44.7%) [26], [27], [28]. Ethnic differences, such as higher LA-GLS values in Asian populations, may partly explain this variation [12]. Age differences may also, as studies with lower GLS values typically included older participants. Variation in LA contouring approaches may influence GLS. CVI42 provides epi-, endo- and global LA-GLS measurements, while Medis and TrufiStrain compute endocardial strain only. To ensure comparability, we used endocardial values across all software platforms. In addition, temporal and spatial cine image resolution has been shown to influence strain measurements, potentially contributing to the heterogeneity in published LA-GLS results [14]. For reliable analysis, temporal resolution ≥ 30 phases and spatial resolution ≤1.8 ×1.8 ×8 mm have been recommended [14].

### 4.3 Impact of field strength

LA-GLS measurements were comparable between 1.5T and 3T, consistent with recent meta-analytic data [12]. However, 3T scans are more prone to flow-related artifacts, such as those near the pulmonary vein ostia [10]. These artifacts can impair tracking and might result in higher exclusion rates. Future studies should therefore account for higher exclusion rates in their power analyses, if they plan to include LA-Strain analysis at 3T.

### 4.4 Inter- and intra-site reproducibility

To our knowledge, this is the first study using traveling volunteers to assess inter-site reproducibility of CMR-FT derived LA-GLS. All GLS components demonstrated excellent agreement across sites and field strengths when analyzed using the same software and version. Intra-site and intra-reader reproducibility was also high. These findings provide a strong foundation for further multi-site and longitudinal studies using LA-GLS.

### 4.5 Subject-related confounders: age

Age significantly influenced LA-GLS, consistent with previous studies [8], [15], [16], [29], [31]. The age dependency of LA-GLS is most likely driven by cellular and structural changes associated with aging [16], [32]. With advancing age, studies demonstrated a progressive loss of cardiomyocytes accompanied by reactive hypertrophy and replacement with fibrotic tissue [33], [34]. These remodeling processes increase myocardial stiffness, reduce compliance, and impair relaxation in both ventricles and atria [33], [35]. Consequently, decreased LA compliance is reflected by reduced reservoir function [11], [16]. In addition, LV diastolic dysfunction and LA stiffness may further impair conduit function [36], [37]. The observed increase in contractile strain with age could represent a compensatory mechanism to maintain atrial output despite these structural changes [16], [32]. However, one meta-analysis found no significant age effect [12]. Two aspects may explain this discrepancy: 1) The pooled mean age in the meta-analysis was higher (49.6 years vs. 39 years), possibly underrepresenting younger individuals. 2) In our study, age-related correlations were strongest in CVI42. Greater variability in Medis measurements may masked age effects, especially when pooled. Age-adjusted normal value ranges were presented in this paper. It is important to note that these values may be influenced by the specific software version and are potentially only applicable to the reported versions.

### 4.6 Subject-related confounders: sex

Consistent with prior findings, LA-GLS did not differ between sexes in CVI42 or Medis. [12], [26], [29] TrufiStrain, however, showed higher reservoir and conduit GLS in females. [15] Whether this reflects a true biological difference, a Type I error or vendor-specific assumptions remains unclear. At present, we do not propose sex-specific normal values, given the lack of sex-related influence observed with the other software vendors.

### 4.7 Subject-related confounders: heart rate

An association between HR and conduit GLS was observed. However, most participants had HRs between 60 and 80 bpm, and extremer values were underrepresented. The observed correlation may have been influenced by outliers, warranting further investigation.

### 4.8 Image acquisition

GLS measurements in this study were based on 2D images, which may be limited by through-plane motion and the complex 3D geometry of the left atrium [10], [14]. Three-dimensional tracking may offer improved accuracy and reproducibility, but remains underexplored [10], [38]. Further research is needed to assess the clinical utility of 3D tissue tracking methods.

## 5 Limitations

This analysis used retrospectively gathered data from prospectively acquired single- center studies. A future prospective multi-center study, including different scanner vendors should be conducted to evaluate the generalizability of our results. Additionally, only healthy controls and AF patients were included, limiting generalizability of this study results. Participant numbers were not balanced between 1.5T and 3T scanners, potentially introducing bias. Furthermore, as the BER-CMR network uses only Siemens scanners, findings cannot be generalized to other vendors. Although we analyzed a subgroup of traveling volunteers, the sample size was small. The small number may mask potential differences. The assessment of potential minor differences between measurements at different sites should be addressed in future, larger-scale studies. All software demonstrated good diagnostic accuracy in distinguishing healthy individuals from patients with atrial fibrillation. However, before software-specific normal values can be implemented into clinical workflows, diagnostic accuracy needs to be evaluated in diseases beyond atrial fibrillation. Strain analysis was based on two-dimensional cine images which is associated with limitations such as through-plane motion [10]. Three-dimensional imaging may improve representation of the complex LA geometry and deformation [10], [14], [38]. However, experience with three-dimensional tracking techniques is limited and should be focus of future research [10], [38]. Patients in the AF group had to be in sinus rhythm throughout the duration of the scan and future studies should evaluate the diagnostic precession of prospective gated or real-time cine images.

## 6 Conclusion

This study highlights the significant impact of post-processing software and age on CMR-FT-derived LA-GLS, underscoring the need for age- and software-specific reference values. While software-related differences remain a major limitation, LA-GLS shows excellent reproducibility and good diagnostic accuracy when the same software, version, and imaging parameters are used. These findings support the use of LA-GLS in multi-center studies and provide a benchmark for future standardization efforts in atrial strain analysis.

## Funding

L.D.K. and M.F. receive funding from the 10.13039/501100005971German Heart Foundation. J.G. receives funding from the DZHK (German Centre for Cardiovascular Research. R.F.T. receives funding from the DZHK, German Heart Foundation and DFG (German Research Foundation). J.S.M. holds institutional grants from the Charité University Medicine Berlin, Germany. T.C. is an employee of Siemens Healthineers. None of the funding interfered with the research or was influenced by them. All other authors have reported that there are no relationships relevant to the content of this publication that are required to be disclosed.

## Author contributions

**Leo Dyke Krüger:** Writing – review & editing, Writing – original draft, Visualization, Validation, Supervision, Software, Project administration, Methodology, Investigation, Formal analysis, Data curation, Conceptualization. **Leonhard Grassow:** Writing – review & editing, Visualization, Validation, Project administration, Investigation, Formal analysis, Data curation. **Groschel Jan Gröschel:** Writing – review & editing, Visualization, Validation, Supervision, Data curation. **Johanna Kuhnt:** Writing – review & editing, Formal analysis, Data curation. **Edyta Blaszczyk:** Writing – review & editing, Validation, Supervision, Data curation. **Maximilian Müller:** Writing – review & editing, Formal analysis, Data curation. **Ralf Felix Trauzeddel:** Writing – review & editing, Supervision, Data curation. **Teodora Chitiboi:** Writing – review & editing, Validation, Supervision, Software. **Jeanette Schulz-Menger:** Writing – review & editing, Validation, Supervision, Software, Resources, Project administration, Methodology, Funding acquisition, Conceptualization. **Maximilian Fenski:** Writing – review & editing, Writing – original draft, Visualization, Validation, Supervision, Software, Resources, Project administration, Methodology, Investigation, Funding acquisition, Formal analysis, Data curation, Conceptualization.

## Ethics approval and consent

Data collection and participant recruitment were authorized by the ethics committee of Charité – Universitätsmedizin Berlin, Germany (EA 1 253 21), and conducted in accordance with institutional guidelines and the principles of the Declaration of Helsinki. Written informed consent was obtained from all participants before study enrollment.

## Declaration of competing interests

The authors declare the following financial interests/personal relationships which may be considered as potential competing interests. Leo Dyke Krueger reports a relationship with German Heart Foundation that includes: funding grants. Maximilian Fenski reports a relationship with German Heart Foundation that includes: funding grants. Ralf Felix Trauzeddel reports a relationship with German Heart Foundation that includes: funding grants. Ralf Felix Trauzeddel reports a relationship with German Centre for Cardiovascular Research that includes: funding grants. Ralf Felix Trauzeddel reports a relationship with German Heart Foundation that includes: funding grants. Teodora Chitiboi reports a relationship with Siemens Healthineers AG that includes: employment. Jeanette Schulz-Menger reports a relationship with Charité University Hospital Berlin that includes: funding grants. The remaining authors declare that they have no known competing financial interests or personal relationships that could have appeared to influence the work reported in this paper.

## Data Availability

Due to local data protection laws, public access is not possible. Data may be available from the corresponding author upon reasonable request.

## Footnotes

Appendix A Supplementary data associated with this article can be found in the online version at doi:10.1016/j.jocmr.2026.102692.

## Footnotes

1 Available for research purposes only. Not commercially available. Future commercial availability cannot be guaranteed.

## References

[1] Serenelli M., Cantone A., Dal P.B., Di I.L., Fiorio A., Pavasini R. (2023). Atrial longitudinal strain predicts new-onset atrial fibrillation. JACC Cardiovasc Imaging Am Coll Cardiol Found.

[2] Akintoye E., Majid M., Klein A.L., Hanna M. (2023). Prognostic utility of left atrial strain to predict thrombotic events and mortality in amyloid cardiomyopathy. JACC Cardiovasc Imaging Am Coll Cardiol Found.

[3] Moon M.-G., Hwang I.-C., Lee H.-J., Kim S.-H., Yoon Y.E., Park J.-B. (2022). Reverse remodeling assessed by left atrial and ventricular strain reflects treatment response to Sacubitril/Valsartan. JACC Cardiovasc Imaging Am Coll Cardiol Found.

[4] Raafs A.G., Vos J.L., Henkens M.T.H.M., Slurink B.O., Verdonschot J.A.J., Bossers D. (2022). Left atrial strain has superior prognostic value to ventricular function and delayed-enhancement in dilated cardiomyopathy. JACC Cardiovasc Imaging Am Coll Cardiol Found.

[5] Marwick T.H., Chandrashekhar Y. (2024). What is new with understanding the left atrium and what it can tell us. JACC Cardiovasc Imaging Am Coll Cardiol Found.

[6] Hinojar R., Zamorano J.L., Fernández-Méndez M., Esteban A., Plaza-Martin M., González-Gómez A. (2019). Prognostic value of left atrial function by cardiovascular magnetic resonance feature tracking in hypertrophic cardiomyopathy. Int J Cardiovasc Imaging.

[7] Zhao Y., Song Y., Mu X. (2024). Application of left atrial strain derived from cardiac magnetic resonance feature tracking to predict cardiovascular disease: a comprehensive review. Heliyon.

[8] Zhou D., Yang W., Yang Y., Yin G., Li S., Zhuang B. (2022). Left atrial dysfunction may precede left atrial enlargement and abnormal left ventricular longitudinal function: a cardiac MR feature tracking study. BMC Cardiovasc Disord.

[9] Smiseth O.A., Rider O., Cvijic M., Valkovič L., Remme E.W., Voigt J.-U. Myocardial Strain Imaging: Theory, Current Practice, and the Future. JACC Cardiovasc Imaging [Internet]. 2024 [cited 2024 Oct 30]; 10.1016/j.jcmg.2024.07.011. 39269417

[10] Scatteia A., Baritussio A., Bucciarelli-Ducci C. (2017). Strain imaging using cardiac magnetic resonance. Heart Fail Rev.

[11] Evin M., Cluzel P., Lamy J., Rosenbaum D., Kusmia S., Defrance C. (2015). Assessment of left atrial function by MRI myocardial feature tracking. J Magn Reson Imaging.

[12] Yang W., Xu J., Zhu L., Zhang Q., Wang Y., Zhao S. (2024). Myocardial strain measurements derived from MR feature-tracking. Influ Sex Age Field Strength Vend JACC Cardiovasc Imaging.

[13] Pathan F., Zainal Abidin H.A., Vo Q.H., Zhou H., D’Angelo T., Elen E. (2021). Left atrial strain: a multi-modality, multi-vendor comparison study. Eur Heart J Cardiovasc Imaging.

[14] Schmidt-Rimpler J., Backhaus S.J., Hartmann F.P., Schaten P., Lange T., Evertz R. (2024). Impact of temporal and spatial resolution on atrial feature tracking cardiovascular magnetic resonance imaging. Int J Cardiol.

[15] Qu Y.-Y., Buckert D., Ma G.-S., Rasche V. (2021). Quantitative assessment of left and right atrial strains using cardiovascular magnetic resonance based tissue tracking. Front Cardiovasc Med.

[16] Liao J.-N., Chao T.-F., Kuo J.-Y., Sung K.-T., Tsai J.-P., Lo C.-I. (2017). Age, sex, and blood pressure-related influences on reference values of left atrial deformation and mechanics from a large-scale asian population. Circ Cardiovasc Imaging Am Heart Assoc.

[17] Raisi-Estabragh Z., Szabo L., McCracken C., Bülow R., Aquaro G.D., Andre F. (2024). Cardiovascular magnetic resonance reference ranges from the healthy hearts consortium. JACC Cardiovasc Imaging Am Coll Cardiol Found.

[18] Hindricks G., Potpara T., Dagres N., Arbelo E., Bax J.J., Blomström-Lundqvist C. (2021). 2020 ESC Guidelines for the diagnosis and management of atrial fibrillation developed in collaboration with the European Association for Cardio-Thoracic Surgery (EACTS): the task force for the diagnosis and management of atrial fibrillation of the European Society of Cardiology (ESC) Developed with the special contribution of the European Heart Rhythm Association (EHRA) of the ESC. Eur Heart J.

[19] Gröschel J., Trauzeddel R.-F., Müller M., von Knobelsdorff-Brenkenhoff F., Viezzer D., Hadler T. (2023). Multi-site comparison of parametric T1 and T2 mapping: healthy travelling volunteers in the Berlin research network for cardiovascular magnetic resonance (BER-CMR). J Cardiovasc Magn Reson J Soc Cardiovasc Magn Reson.

[20] Schulz-Menger J., Bluemke D.A., Bremerich J., Flamm S.D., Fogel M.A., Friedrich M.G. (2020). Standardized image interpretation and post-processing in cardiovascular magnetic resonance - 2020 update. J Cardiovasc Magn Reson.

[21] Kawel-Boehm N., Hetzel S.J., Ambale-Venkatesh B., Captur G., Francois C.J., Jerosch-Herold M. (2020). Reference ranges (“normal values”) for cardiovascular magnetic resonance (CMR) in adults and children: 2020 update. J Cardiovasc Magn Reson.

[22] Zhang Y., Mui D., Chirinos J.A., Zamani P., Ferrari V.A., Chen Y. (2021). Comparing cardiovascular magnetic resonance strain software packages by their abilities to discriminate outcomes in patients with heart failure with preserved ejection fraction. J Cardiovasc Magn Reson.

[23] Amzulescu M.S., De Craene M., Langet H., Pasquet A., Vancraeynest D., Pouleur A.C. (2019). Myocardial strain imaging: review of general principles, validation, and sources of discrepancies. Eur Heart J Cardiovasc Imaging.

[24] Zamani S.K., Samuel T.J., Wei J., Thomson L.E.J., Tamarappoo B., Sharif B. (2020).

[25] Singleton M.J., Nelson M.B., Samuel T.J., Kitzman D.W., Brubaker P., Haykowsky M.J. (2022). Left atrial stiffness index independently predicts exercise intolerance and quality of life in older, obese patients with heart failure with preserved ejection fraction. J Card Fail.

[26] Truong V.T., Palmer C., Wolking S., Sheets B., Young M., Ngo T.N.M. (2020). Normal left atrial strain and strain rate using cardiac magnetic resonance feature tracking in healthy volunteers. Eur Heart J Cardiovasc Imaging.

[27] Han P.-L., Shen M.-T., Jiang Y., Jiang Z.-K., Li K., Yang Z.-G. (2023). Prognostic value of left atrial reservoir strain in left ventricular myocardial noncompaction: a 3.0 T cardiac magnetic resonance feature tracking study. J Magn Reson Imaging.

[28] Zhou H., An D.-A., Ni Z., Xu J., Zhou Y., Fang W. (2022). Incremental diagnostic value of CMR-derived LA strain and strain rate in dialysis patients with HFpEF. Eur J Radiol.

[29] Nyberg J., Jakobsen E.O., Østvik A., Holte E., St ølen S., Lovstakken L. (2023). Echocardiographic reference ranges of global longitudinal strain for all cardiac chambers using guideline-directed dedicated views. JACC Cardiovasc Imaging Am Coll Cardiol Found.

[31] Singh A., Carvalho Singulane C., Miyoshi T., Prado A.D., Addetia K., Bellino M. (2022). Normal values of left atrial size and function and the impact of age: results of the world alliance societies of echocardiography study. J Am Soc Echocardiogr.

[32] Boyd A.C., Schiller N.B., Leung D., Ross D.L., Thomas L. (2011). Atrial dilation and altered function are mediated by age and diastolic function but not before the eighth decade. JACC Cardiovasc Imaging.

[33] Olivetti G., Melissari M., Capasso J.M., Anversa P. (1991). Cardiomyopathy of the aging human heart. Myocyte loss and reactive cellular hypertrophy. Circ Res Am Heart Assoc.

[34] Horn M.A., Trafford A.W. (2016). Aging and the cardiac collagen matrix: novel mediators of fibrotic remodelling. J Mol Cell Cardiol.

[35] Lakatta E.G., Levy D. (2003). Arterial and cardiac aging: major shareholders in cardiovascular disease enterprises. Circ Am Heart Assoc.

[36] Nikitin N.P., Witte K.K.A., Thackray S.D.R., Goodge L.J., Clark A.L., Cleland J.G.F. (2003). Effect of age and sex on left atrial morphology and function. Eur J Echocardiogr.

[37] Triposkiadis F., Tentolouris K., Androulakis A., Trikas A., Toutouzas K., Kyriakidis M. (1995). Left atrial mechanical function in the healthy elderly: new insights from a combined assessment of changes in atrial volume and transmitral flow velocity. J Am Soc Echocardiogr.

[38] Pedrizzetti G., Claus P., Kilner P.J., Nagel E. (2016). Principles of cardiovascular magnetic resonance feature tracking and echocardiographic speckle tracking for informed clinical use. J Cardiovasc Magn Reson.

