# Supplementary material for: Confounders, diagnostic accuracy, and reproducibility in cardiovascular magnetic resonance-feature tracking-derived left atrial strain: A Berlin Research Network for cardiovascular magnetic resonance multi-software, multi-site comparison
Source: J Cardiovasc Magn Reson. 2026 Jan 15;28(1):102692. doi: 10.1016/j.jocmr.2026.102692 (PMC13196062; doi:10.1016/j.jocmr.2026.102692)
Supplement: Supplementary file 1 — Supplementary material [file mmc1.docx]

**Supplemental Data**

[Supplemental Methods 1 – bSSFP Acquisition Parameters 2](#_Toc216116893)

[Supplemental Methods 2 - Strain Analysis Protocoll 3](#_Toc216116894)

[Supplemental Table 1 - Results of Mixed Model with Repeated Measures before Backwards Selection 4](#_Toc216116895)

[Supplemental Table 2 - Results of Mixed Model with Repeated Measures before Backwards Selection for Conduit Strain 5](#_Toc216116896)

[Supplemental Table 3 - Results of Mixed Model with Repeated Measures before Backwards Selection for Contractile Strain 6](#_Toc216116897)

[Supplemental Table 4 - Results of Mixed Model with Repeated Measures after Backwards Selection 7](#_Toc216116898)

[Supplemental Table 5 - Results of ROC Analysis 8](#_Toc216116899)

[Supplemental Tabel 6 - Baseline characteristics and results of LA-GLS measurements across software platforms for the diagnostic accuracy analysis. 9](#_Toc216116900)

[Supplemental Table 7 - Clinical characteristics of AF patients 11](#_Toc216116901)

[Supplemental Table 8 - Left ventricular and atrial size and function of AF patients 12](#_Toc216116902)

[Supplemental Figure 1 – Examples for exclusion due to unsatisfactory tracking quality 13](#_Toc216116903)

[Supplemental Figure 2 – Comparison between CVI42 and Trufistrain 14](#_Toc216116904)

[Supplemental Figure 3 – Comparison between CVI and Medis 16](#_Toc216116905)

[Supplemental Figure 4 – Comparison between TrufiStrain and Medis 18](#_Toc216116906)

[Supplemental Figure 5 – Inter-Centre Comparison for Reservoir Strain 20](#_Toc216116907)

[Supplemental Figure 6 – Inter-Centre Comparison for Conduit Strain 22](#_Toc216116908)

[Supplemental Figure 7 – Inter-Centre Comparison for Contractile Strain 24](#_Toc216116909)

[Supplemental Figure 8 - Intra-Center Comparison 26](#_Toc216116910)

[Supplemental Figure 9 - LA Strain Age Group Comparison 28](#_Toc216116911)

[Supplemental Figure 10 - LA Strain and correlation with Heart Rate 29](#_Toc216116912)

[Supplemental Figure 11 - Subgroup Analysis: Software Comparison 31](#_Toc216116913)

[Supplemental Figure 12 – Subgroup analysis: Correlation between LA Strain and Age 33](#_Toc216116914)

[Supplemental Figure 13 – Subgroup Analysis: Sex Differences in LA strain 34](#_Toc216116915)

[Supplemental Figure 14 – Subgroup Analysis: Correlation between LA Strain and HR 36](#_Toc216116916)

# Supplemental Methods 1 – bSSFP Acquisition Parameters

Left atrial strain analysis was conducted using balanced steady-state free precession (bSSFP) CMR cine imaging in two long-axis views (2-chamber view and 4-chamber view). Sequence parameters were as follows:

At 1.5 Tesla scanners (Avanto and AvantoFit, Siemens Healthnieers, Erlangen, Germany): repetition time: 34.7 - 62 ms, echo time: 1.14 - 1.48 ms, flip angle: 70 - 80 degrees, fields of view: 255 - 308.75 x 340 - 380 mm2, matrix: 144 - 216 x 192 - 256, slice thickness and gap: 7 / 0 mm and 7 / 3 mm, 30 cardiac phases.

At 3 Tesla scanners (SkyraFit and Verio, Siemens Healthineers, Erlangen, Germany): repetition time: 3.1 - 45.78 ms, echo time: 1.3 - 1.43 ms, flip angle: 45 - 54 degrees, fields of view: 255 - 301.15 x 340 - 360 mm2, matrix: 156 - 174 x 192 - 208, slice thickness and gap: 7 / 0 mm, 6 / 0 mm and 7 / 3 mm, 30 cardiac phases.

# Supplemental Methods 2 - Strain Analysis Protocol

Strain analysis was performed manually according to a strict procedure: Contours in CVI and Siemens were drawn in the first phase, usually equivalent to the atrial end-systole (aES). In Medis aES and atrial end-diastole (aED) were visually identified and contoured. aES was defined as the smallest atrial area, while aED was defined as the largest atrial area. Contours were drawn along the subendocardial border of the LA in the two- and four-chamber view. In addition, CVI strain analysis required contouring of the epicardial border, offering both endocardial and epicardial strain. To enable better comparison between software, only endocardial strain analysis was used in this study. Reference frame adjustments for CVI were done for cases, in which the first frame was not equvilent to the atrial end-systole. *Figure 1* shows exemplary contouring and left atrial strain curves for each software vendor. Particular attention was paid to the correct position on the valve annulus. Pulmonary veins and LA appendage were excluded, and the boarders extrapolated. For each case, visual evaluation of tracking was conducted and adjusted if needed.

| **Supplemental Table 1 - Results of Mixed Model with Repeated Measures before Backwards Selection** | | | |
| --- | --- | --- | --- |
|  |  | F-value | PR > F |
| Reservoir | Software | 452.66 | <.001 |
|  | Field strength | 0.02 | .886 |
|  | scanner | 0.43 | .829 |
|  | Sex | 7.92 | .014 |
|  | Age | 6.19 | .004 |
|  | BMI | 0.12 | .640 |
|  | HR | 1.97 | .084 |
|  | RR_sys_ | 0.38 | .518 |
|  | RR_dia_ | 0.07 | .315 |
| *RR_sys_ = systolic blood pressure ; RR_dia_ = diastolic blood pressure* | | | |

| **Supplemental Table 2 - Results of Mixed Model with Repeated Measures before Backwards Selection for Conduit Strain** | | | |
| --- | --- | --- | --- |
|  |  | F-value | PR > F |
| Conduit | Software | 255.67 | <.001 |
|  | Field strength | 0.27 | .590 |
|  | scanner | 0.49 | .627 |
|  | Sex | 18.31 | <.001 |
|  | Age | 30.21 | <.001 |
|  | BMI | 0.82 | .458 |
|  | Heart rate | 18.54 | <.001 |
|  | RR_sys_ | 0.28 | .500 |
|  | RR_dia_ | 0.29 | .899 |
| *RR_sys_ = systolic blood pressure ; RR_dia_ = diastolic blood pressure* | | | |

| **Supplemental Table 3 - Results of Mixed Model with Repeated Measures before Backwards Selection for Contractile Strain** | | | |
| --- | --- | --- | --- |
|  |  | F-value | PR > F |
| Contractile | Software | 210.57 | <.001 |
|  | Field strength | 11.54 | .669 |
|  | Scanner | 0.23 | .973 |
|  | Sex | 0.37 | .534 |
|  | Age | 0.31 | .480 |
|  | BMI | 0.18 | .826 |
|  | Heart rate | 3.54 | .014 |
|  | RR_sys_ | 0.08 | .985 |
|  | RR_dia_ | 0.59 | .149 |
| *RR_sys_ = systolic blood pressure ; RR_dia_ = diastolic blood pressure* | | | |

| **Supplemental Table 4 - Results of Mixed Model with Repeated Measures after Backwards Selection** | | | | | |
| --- | --- | --- | --- | --- | --- |
|  |  | F-value | PR > F | Effect size | Confidence interval |
| Reservoir | Software | 452.66 | <.001 | 30.75 | 28.15 to 33.36 |
|  | Sex | 7.92 | .024 | 1.9 | 0.26 to 3.54 |
|  | Age | 6.19 | <.001 | -0.11 | -0.17 to -0.052 |
| Conduit | Software | 255.67 | <.001 | 6.29 | 1.37 to 11.22 |
|  | Sex | 18.31 | <.001 | 2.63 | 1.31 to 3.95 |
|  | Age | 30.21 | <.001 | -0.15 | -0.19 to -0.10 |
|  | Heart rate | 18.54 | <.001 | 0.15 | 0.08 to 0.21 |
| Contractile | Software | 210.57 | <.001 | 19.61 | 14.55 to 24.67 |
|  | Heart rate | 7.81 | .006 | -0.06 | -0.14 to 0.01 |

| Supplemental Table 5 -  Results of ROC Analysis | | | | | |
| --- | --- | --- | --- | --- | --- |
| Component | Software | AUC [95% CI] | Optimal Cutoff | Sensitivity | Specificity |
| **Reservoir** | CVI | 0.81 [0.69–0.90] | 18,32 | 0,7 | 0,9 |
|  | Trufi | 0.76 [0.64–0.88] | 19,01 | 0,63 | 0,9 |
|  | Medis | 0.84 [0.72–0.94] | 27,72 | 0,66 | 1 |
| **Conduit** | CVI | 0.64 [0.48–0.78] | 10,91 | 0,55 | 0,75 |
|  | Trufi | 0.76 [0.63–0.87] | 6,3 | 0,65 | 0,9 |
|  | Medis | 0.82 [0.69–0.92] | 12,24 | 0,66 | 0,95 |
| **Contractile** | CVI | 0.83 [0.72–0.92] | 8,65 | 0,68 | 0,95 |
|  | Trufi | 0.79 [0.66–0.91] | 10,58 | 0,63 | 0,9 |
|  | Medis | 0.80 [0.67–0.91] | 15,06 | 0,66 | 0,9 |
| Youden’s index was used to determine the optimal threshold, along with its corresponding sensitivity and specificity, for each software and strain component in differentiating healthy individuals from patients with atrial fibrillation. | | | | | |

| Supplemental Tabel 6 -  Baseline characteristics and results of LA-GLS measurements across software platforms for the diagnostic accuracy analysis. | | | |
| --- | --- | --- | --- |
|  | Healthy (n=20) | AF (n=40) | P |
| Age, years | 61.8±5.3 | 67.9±8.4 | .005 |
| Male, n (%) | 7/20 (35) | 26/40 (65) | .028 |
| Persistent AF, n(%) | 0 (0) | 19 (48) | N/A |
| BMI, kg/m^2^ | 25.3±3.2 | 28.6±4.2 | .003 |
| Heart rate, bpm | 67±9 | 66±12 | .822 |
| Median CHA2DS2-Vasc Score (IQR) | N/A | 3 (2-4) | N/A |
| Median days since AF diagnosis (IQR) | N/A | 112 (62-338) | N/A |
| LV EDV-I, ml/m^2^ | 75.5±11.2 | 87.7±17.7 | .007 |
| LVEF, % | 64.4±5.2 | 60.7±8.3 | .075 |
| LV Mass-I, g/m^2^ | 52.9±9.9 | 52.2±12.9 | .837 |
| LAV-I ml/m^2^ | 32.7±9.4 | 52.0±15.8 | <.001 |
| **LA-Global longitudinal strain (LA-GLS)** | | | |
| Reservoir Strain, %  CVI | 21.5±3.3 | 16.0±5.2 | <.001 |
| Reservoir Strain, %  TrufiStrain | 26.2±8.4 | 17.4±9.1 | <.001 |
| Reservoir Strain, %  Medis | 43.8±13.7 | 26.8±12.6 | <.001 |
| Contractile Strain, % CVI | 11.0±1.5 | 6.4±4.1 | <.001 |
| Contractile Strain, % TrufiStrain | 16.4±6.7 | 9.3±6.3 | <.001 |
| Contractile Strain, % Medis | 24.0±11.7 | 12.3±9.1 | <.001 |
| Conduit Strain, %  CVI | 12.9±4.8 | 10.8±4.1 | .077 |
| Conduit Strain, %  TrufiStrain | 9.7±3.4 | 6.3±4.8 | .005 |
| Conduit Strain, %  Medis | 19.8±6.5 | 11.6±7.1 | <.001 |

| Supplemental Table 7 -  Clinical characteristics of AF patients | | |
| --- | --- | --- |
|  | Px-AF (N=21) | Ps-AF (N=19) |
| Age, years | 71.0 (25.4-76.0) | 69.0 (62.0-71.0) |
| Male, n (%) | 8 (38) | 18 (95) |
| BMI, kg/m^2^ | 28.4 (25.4-31.0) | 29.4 (25.2-33.1) |
| Heart rate, bpm | 61±9.4 | 71.9 ± 11.5 |
| CHA2DS2-Vasc Score | 3 (2-4) | 2 (1-3) |
| Median days since AF diagnosis (IQR) | 87 (61-198) | 168 (97-613) |
| Diabetes, n (%) | 6 (29) | 2 (11) |
| Hypertension, n (%) | 15 (75) | 14 (74) |
| Hyperlipidemia, n (%) | 10 (48) | 6 32) |
| Heart failure, n (%) | 2 (10) | 2 11) |
| CAD, n (%) | 5 (24) | 3 (16) |
| Stroke, TIA or arterial thromboembolism, n (%) | 3 (14) | 3 (16) |
| Peripheral vascular disease, n (%) | 4 (19) | 4 (21) |
| Clinical characteristics of AF patients according to AF-type, Values are shown as mean±SD or median (IQR), Px-AF = patients with paroxysmal AF, Ps-AF = patients with persistent AF. AF = Atrial Fibrillation. | | |

| Supplemental Table 8 -  Left ventricular and atrial size and function of AF patients | | |
| --- | --- | --- |
|  | Px-AF (N=21) | Ps-AF (N=19) |
| LV EDV-I, ml/m^2^ | 82.3 ± 17.9 | 86.8±19.1 |
| LVEF, % | 59.7±7.7 | 56.6±7.3 |
| LV Mass-I, g/m^2^ | 45.4 ± 10.8 | 49.6±11.2 |
| LAV-I ml/m^2^ | 45.1±9.4 | 59.3±18.0 |
| LAEF, % | 51.1 ± 11.7 | 36.2±14.1 |
| LA LGE, % | 1.16 [0.43-2.27] | 3.65 [1.84-7.16] |
| Left ventricular and atrial size and function of AF patient, Values are shown as mean±SD or median (IQR), Px-AF = patients with paroxysmal AF, Ps-AF = patients with persistent AF. AF = Atrial Fibrillation. | | |


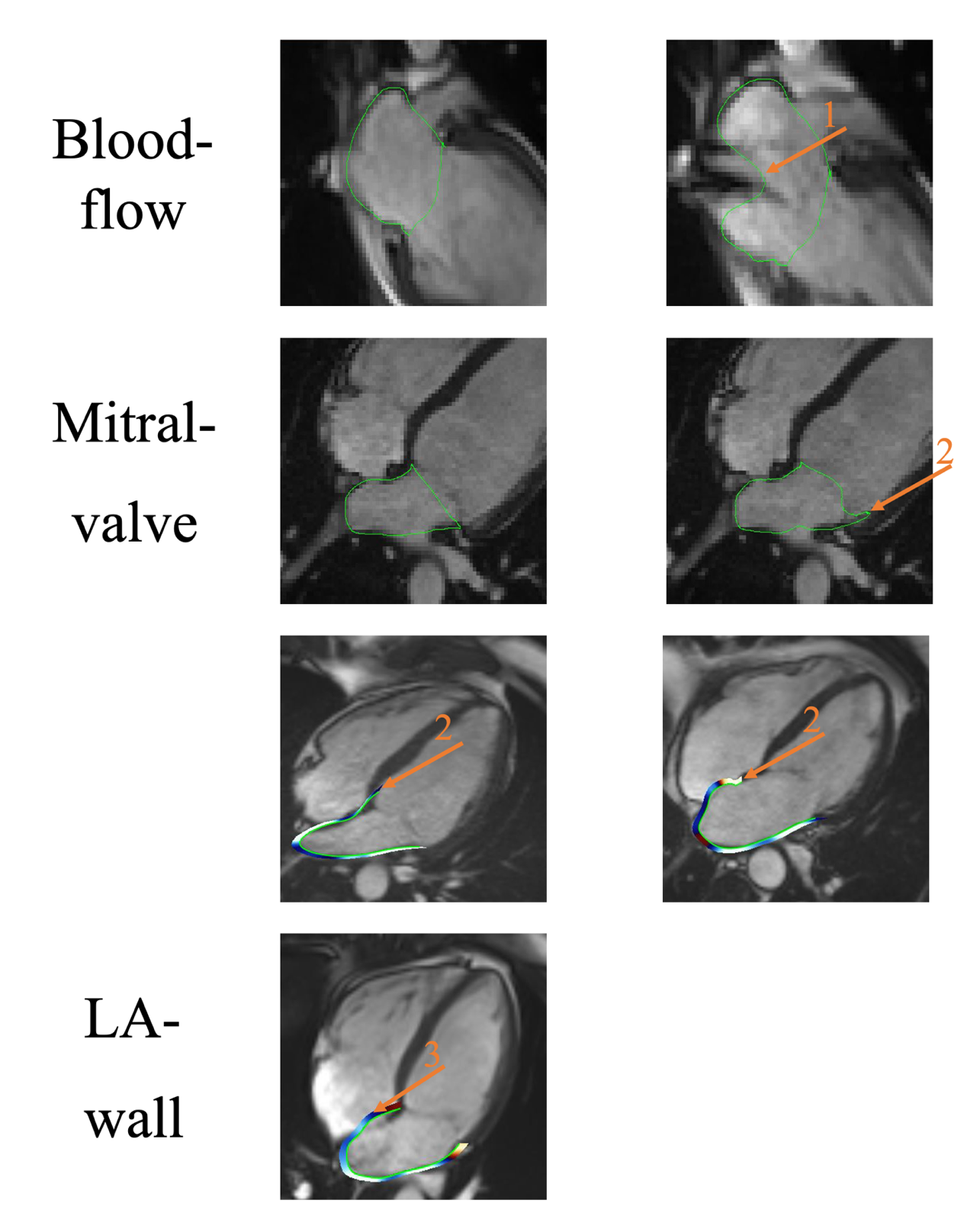


# Supplemental Figure 1 – Examples for exclusion due to unsatisfactory tracking quality

Examples in which adjustment failed to achieve acceptable tracking, leading to exclusion. Orange arrows point to erroneous tracking. Faulty tracking of pulmonary vein inflow (1)[CVI42 n = 2; TrufiStrain n = 7; Medis n = 5]. Contour failed to track mitral valve anulus (2)[TrufiStrain n = 7 ; Medis n = 11].





# Supplemental Figure 2 – Comparison between CVI42 and Trufistrain

Bland-Altman Plots to assess bias between CVI42 and TrufiStrain for (A) reservoir strain , (B) conduit strain and (C) contractile strain. The middle line represents the bias. The Orange box represents the 95% range of agreement.

LL = lower limit of agreement ; UL = upper limit of agreement.





# Supplemental Figure 3 – Comparison between CVI and Medis

Bland-Altman Plots to assess bias between CVI42 and Medis for (A) reservoir strain , (B) conduit strain and (C) contractile strain. The middle line represents the bias. The Orange box represents the 95% range of agreement.

LL = lower limit of agreement ; UL = upper limit of agreement.

*

*

# Supplemental Figure 4 – Comparison between TrufiStrain and Medis

Bland-Altman Plots to assess bias between TrufiStrain and Medis for (A) reservoir strain , (B) conduit strain and (C) contractile strain. The middle line represents the bias. The Orange box represents the 95% range of agreement.

LL = lower limit of agreement ; UL = upper limit of agreement.

*

*

# Supplemental Figure 5 – Inter-Centre Comparison for Reservoir Strain

Bland-Altman Plots comparing left atrial reservoir strain values between centers 1, 2, 3 and 4.

The middle line represents the bias. The orange box represents the 95% range of agreement. 1 = Centre 1 (1.5T) ; 2 = Centre 2 (3T) ; 3 = Centre 3 (3T) ; 4.1 = Centre 4 (3T) ; 4.2 = Re-scan at Centre 4 (3T). LL = lower limit of agreement ; UL = upper limit of agreement.





# Supplemental Figure 6 – Inter-Centre Comparison for Conduit Strain

Bland-Altman Plots comparing left atrial conduit strain values between centers 1, 2, 3 and 4.

Orange box represents the 95% range of agreement.

1 = Centre 1 (1.5T) ; 2 = Centre 2 (3T) ; 3 = Centre 3 (3T) ; 4.1 = Centre 4 (3T) ; 4.2 = Re-scan at Centre 4 (3T). LL = lower limit of agreement ; UL = upper limit of agreement.





# Supplemental Figure 7 – Inter-Centre Comparison for Contractile Strain

Bland-Altman Plots comparing left atrial contractile strain values between centers 1, 2, 3 and 4.

Orange box represents the 95% range of agreement.

1 = Centre 1 (1.5T) ; 2 = Centre 2 (3T) ; 3 = Centre 3 (3T) ; 4.1 = Centre 4 (3T) ; 4.2 = Re-scan at Centre 4 (3T). LL = lower limit of agreement ; UL = upper limit of agreement.





# Supplemental Figure 8 - Intra-Center Comparison

Bland-Altman Plots to compare (A) reservoir strain, (B) conduit strain and (C) contractile Strain at two different time points at the same center. The middle line represents the bias. The orange box represents the 95% range of agreement. Measurements presented in this graph were conducted using CVI42.

CI = Confidence intervall ; LL = lower limit of agreement ; UL = upper limit of agreement.





# Supplemental Figure 9 - LA Strain Age Group Comparison

Age group comparison for reservoir strain (orange) between CVI42 (A), TrufiStrain (B) and Medis(C). Age group comparison for conduit strain (purple) between CVI42 (E), TrufiStrain (F) and Medis (G). Age group comparison for contractile strain (red) between CVI42 (H), TrufiStrain (I) and Medis (J).

Box and Whisker Plots with box showing mean and interquartile range and whisker presenting minimum and maximum values.

** p < .010; **** p < .001





# Supplemental Figure 10 - LA Strain and correlation with Heart Rate

Correlation between Left atrial strain, assessed in CVI42 (A), a TrufiStrain (B) and Medis (C). Reservoir strain is shown in orange. Conduit Strain is shown in purple. Contractile Strain is shown in red.

Bpm = beats per minute ; HR = Heart rate ; ρ = Spearman Correlation Coefficient


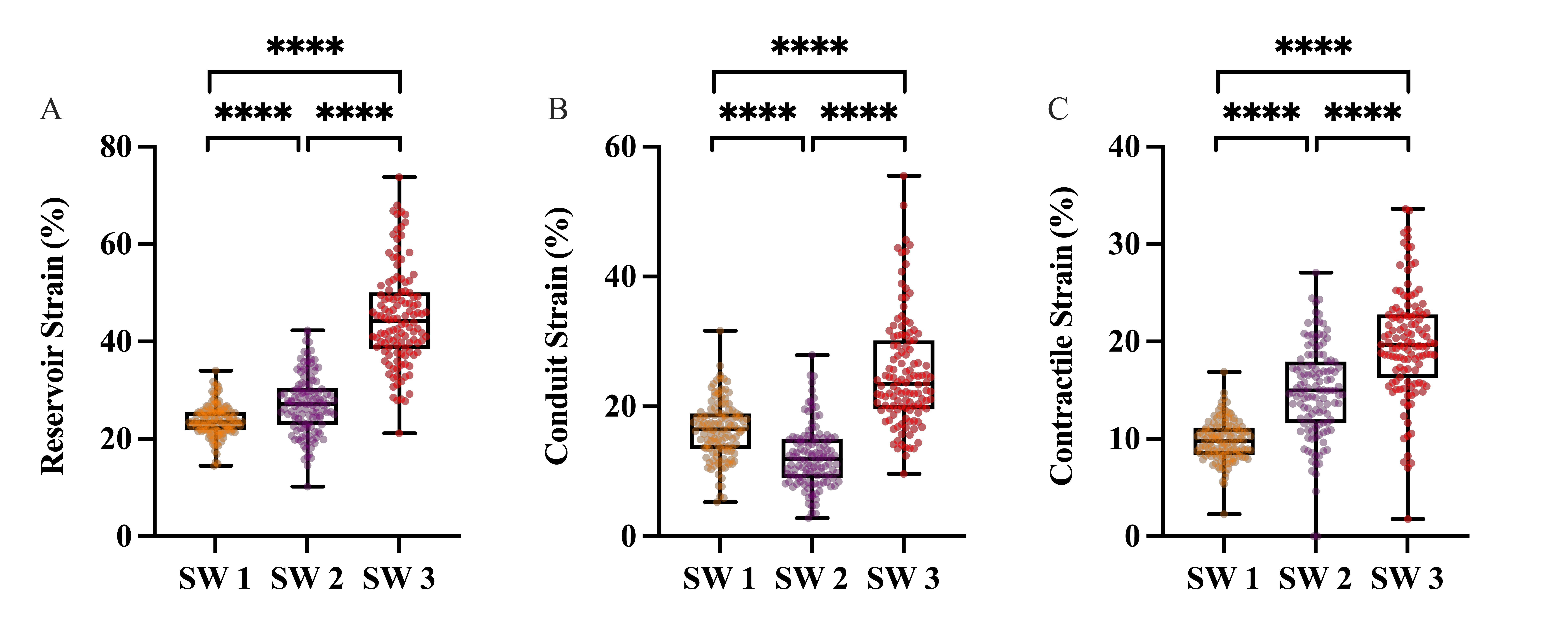


# Supplemental Figure 11 - Subgroup Analysis: Software Comparison

Subgroup, which only included cases with sufficient tracking quality across all three software vendors (n = 122). (A) Left atrial reservoir strain (B) Left atrial conduit strain (C) Left atrial contractile strain. Data for CVI42 is shown in orange, for the TrufiStrain Research Prototype in purple and for Medis in red.

Box and Whisker Plots with boxes showing mean and interquartile range and whisker presenting minimum and maximum values.

**** = p < .001 ; SW 1 = CVI42 ; SW2 = TrufiStrain Research Prototype ; SW 3 = Medis





# Supplemental Figure 12 – Subgroup analysis: Correlation between LA Strain and Age

Correlation analysis for subgroup, which only included cases with sufficient tracking quality across all three software vendors (n = 122). (A) Left atrial strain according to age for CVI42 (B) Left atrial strain according to age for TrufiStrain Research Prototype (C) Left atrial strain according to age for Medis. Reservoir strain is shown in orange. Conduit Strain is shown in purple. Contractile Strain is shown in red.

ρ = Spearman Correlation Coefficient ; SW 1 = CVI42 ; SW 2 = TrufiStrain Research Prototype ; SW 3 = Medis

**
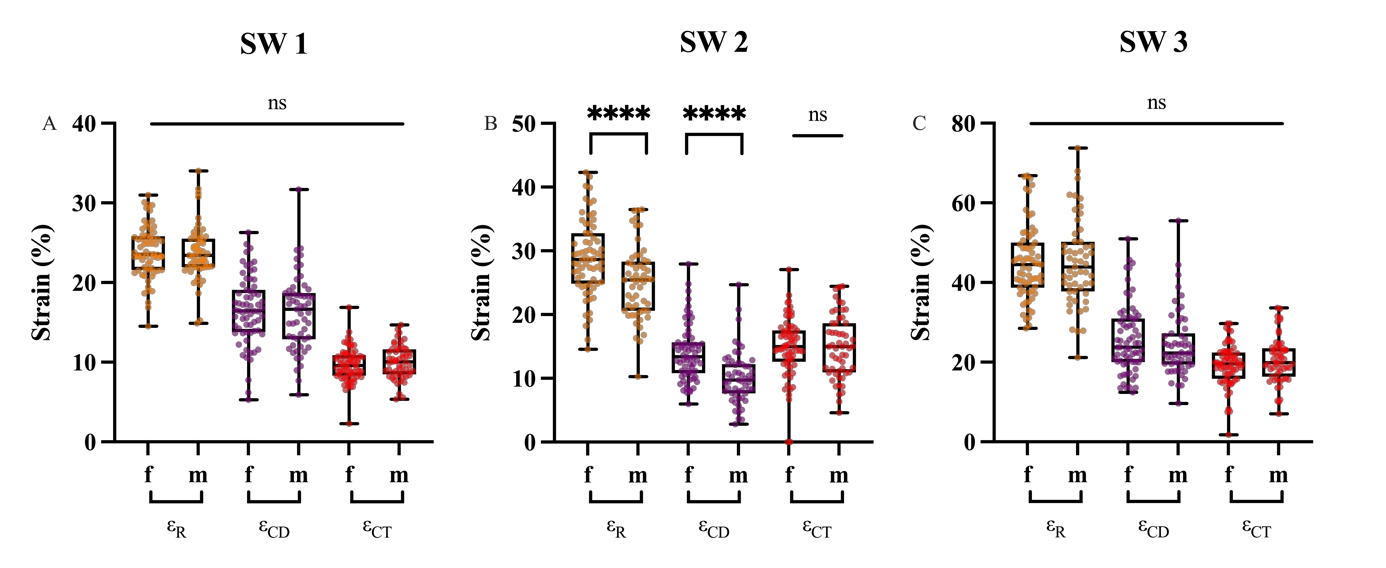
**

# Supplemental Figure 13 – Subgroup Analysis: Sex Differences in LA strain

Sex comparison for subgroup of n = 122 for each strain component using CVI42 (A), . TrufiStrain Research Prototype (B) and Medis (C). Box and Whisker Plots with boxes showing mean and interquartile range and whiskers representing minimum and maximum values.

**** = p < .001 ; ε_R_ = Reservoir Strain ; ε_CD_ = Conduit Strain ; ε_CT_ = Contractile Strain ; f = female ; m = male ; ns = not significant ; SW 1 = CVI42 ; SW 2 = TrufiStrain Research Prototype ; SW 3 = Medis.

**

**

# Supplemental Figure 14 – Subgroup Analysis: Correlation between LA Strain and HR

Correlation Analysis for subgroup, which only included cases with sufficient tracking quality across all three software vendors (n = 122). (A) Left atrial strain according to age for CVI42 (B) Left atrial strain according to age for TrufiStrain (C) Left atrial strain according to age for Medis. Reservoir strain is shown in orange. Conduit Strain is shown in purple. Contractile Strain is shown in red.

Bpm = beats per minute ; HR = Heart rate ; ρ = Spearman Correlation Coefficient
